# Single-dose and multiple-dose pharmacokinetics of zaltoprofen after oral administration in healthy Chinese volunteers

**DOI:** 10.1016/S1674-8301(11)60007-9

**Published:** 2011-01

**Authors:** Lingjun Li, Pengcheng Ma, Yuping Cao, Lei Tao, Yue Tao

**Affiliations:** Department of Pharmacology, Institute of Dermatology, Chinese Academy of Medical Sciences and Peking Union Medical College, Nanjing, Jiangsu 210042, China

**Keywords:** zaltoprofen, nonsteroidal anti-inflammatory drug, pharmacokinetics

## Abstract

Zaltoprofen, a propionic acid derivative of non-steroidal anti-inflammatory drugs, has strong inhibitory effects on actue and chronic inflammation. A randomized, dose-escalating study was conducted to evaluate the pharmacokinetics of single and multiple oral doses of zaltoprofen in 12 healthy Chinese volunteers. Pharmacokinetics was determined from serial blood samples obtained up to 24 h after administration of a single dose of zaltoprofen at 80, 160 or 240 mg and after multiple doses of zaltqorofen at 80 mg 3 times daily. The C_max_ and AUC_0-24_ of zaltoprofen were found to be proportional to drug dose. Zaltoprofen was rapidly absorbed (t_max_ =1.46±0.83 h) and cleared (t_1/2_ =4.96±2.97 h). Pharmacokinetic parameters after multiple doses were similar to those after single doses. Zaltoprofen was well tolerated. These results support a tid regimen of zaltoprofen for the management of acute and chronic inflammation.

## INTRODUCTION

Zaltoprofen, a propionic acid derivative of non-steroidal anti-inflammatory drug (NSAID), has strong inhibitory effects on acute and chronic inflammation[1]. Zaltoprofen may produce its analgesic effects through a peripheral mechanism and especially for the treatment of inflammatory conditions accompanied by pain[2]. However, the adverse effects of zaltoprofen on gastric and small intestinal mucosa were very weak[3]. In addition, zaltoprofen has more powerful inhibitory effects on bradykinin (BK)-nociception than other NSAIDs[4].

The therapeutic dose of zaltoprofen is about 80-240 mg/d given orally. High performance liquid chromatography with ultraviolet detector (HPLC-UV) is a sensitive determination method for zaltoprofen in human plasma of the elimination phase. Although the pharmacokinetic profile of one single-dose of zaltoprofen at 160 mg in Korean healthy volunteers was studied[5], the pharmacokinetic profiles of zaltoprofen following single and multiple oral doses have not yet been fully characterized in Chinese volunteers. In addition, dose proportionality of the plasma levels over the therapeutic range has not been determined. Therefore, the purpose of this study was to evaluate the pharmacokinetics of zaltoprofen after single escalating oral doses (80-240 mg) and multiple doses (80 mg, 3 times daily) in healthy Chinese volunteers. The effects of gender on the pharmacokinetics of zaltoprofen were also evaluated.

## MATERIALS AND METHODS

### Materials and reagents

Zaltoprofen in human plasma was determined by HPLC. The Shimadzu HPLC system consisted of LC-10ATvp pumps and a SPD-10Avp UV-Vis absorbance detector (Shimadzu), operated at 332 nm, and an N2000 chromatographic workstation (Intelligent Information Institute, Zhejiang University, Hangzhou, China). Liquid chromatographic separation was achieved on a Waters Symmetry C18 column (250 mm, 4.6 mm, 5 µm). The mobile phase consisted of acetonitrile-10 mmol/L ammonium acetate (pH 3.0; 55:45, v/v) pumped at an isocratic flow rate of 1 mL/min.

**Fig.1:**
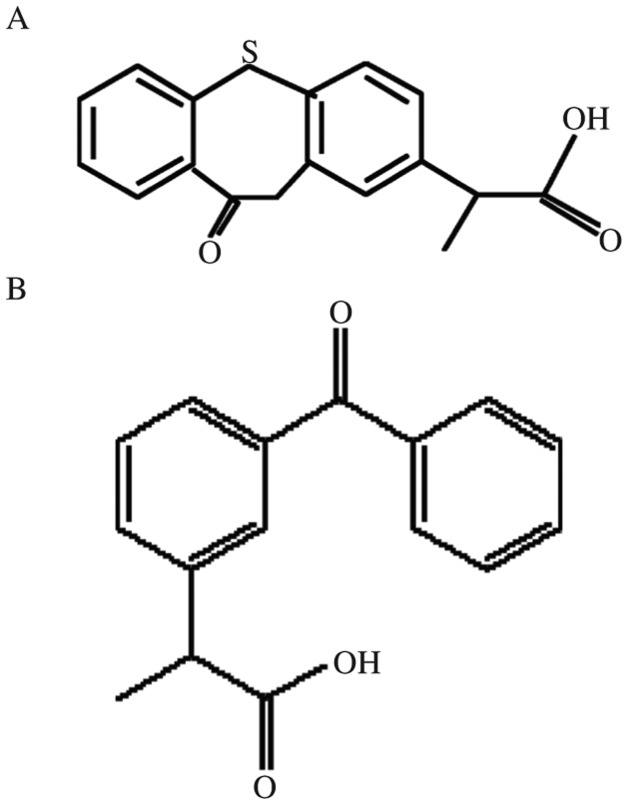
Chemical structure of zaltoprofen (A) and ketoprofen (B)

Zaltoprofen test tablets (Batch No. 080401, 80 mg zaltoprofen), zaltoprofen reference standard (100.0% purity, Batch No. ZPJ-0180707, ***Fig. 1***) were supplied by Jiangsu Feima Pharmaceutical Co. Ltd (Nantong, China); ketoprofen reference standard (internal standard, IS, Batch No.100337-200502, 99.9% purity, ***Fig. 1***) was supplied and identified by Jiangsu Institute for Drug Control (Nanjing, China). Acetonitrile was of chromatographic grade and was purchased from Merck (Germany). Ammonium acetate was of chromatographic grade and was purchased from Tedia (Tedia Company, USA). Other chemicals were all of analytical grade. Water was purified by redistillation before use.

### Subjects

Twelve healthy Chinese volunteers (6 male and 6 female volunteers, aged 22-29 years, body mass index 19.84-23.95 kg/m^2^) were enrolled in the study. All subjects were in good health after the assessment of medical history, physical examination, electrocardiogram and standard laboratory test results (blood cell count, biochemical profile and urinalysis) and a negative pregnancy test result. Subjects were excluded for the following reasons: a history of clinically significant cardiovascular, renal, hepatic, pulmonary, gastrointestinal, hematological or vascular disease; a history of nervous system or a psychiatric disorder; women during breastfeeding or menstruating; a history of alcohol or drug abuse; allergy or hypersensitivity to the study drugs; a positive test result for hepatitis B virus; blood donation within the previous 3 months; participation in another investigational drug study within the previous 3 months; treatment with any drug during the previous 2 w or the entry into the study.

### Study designs

All subjects received in sequential order zaltoprofen orally 80, 160 and 240 mg. All doses were taken in the morning after an overnight fast (10 h). Subjects fasted 4 h after drug administration. Venous blood samples about 3 ml were collected in heparinized tubes at predose (0 h) and 0.25, 0.5, 0.75, 1, 1.5, 2, 3, 4, 6, 8, 10, 12 and 24 h post drug administration. The plasma samples were separated by centrifugation at 4,000 g for 3 min and stored at -20°C until analysis. After the single dose study was finished, all subjects received 80 mg of zaltoprofen 3 times a day for 6 consecutive days. On the seventh day, all subjects received 80 mg zaltoprofen only in the morning and the blood samples were also collected at the above time points. To establish that the steady-state condition had been reached, additional predose blood samples were collected prior to the morning dose on d 5, 6 and 7. In each period, the volunteers stayed in the Clinical Pharmacology Unit. Every period washout interval was 1 week.

The study was performed in accordance with good clinical practice (GCP) guidelines of the State Food and Drug Administration in China and the Declaration of Helsinki. The protocol was reviewed and approved by the Ethics Committee of the Institue of Dermatology, Chinese Academy of Medical Sciences and Peking Union Medical College (Nanjing, China), and all subjects gave a written consent for their participation after having been informed by the medical supervisor about the detailed information of the study.

### Biological samples

Human blank plasma samples for the development, validation and control of the method were obtained from healthy, drug-free volunteer blood donors at the Jiangsu Blood Center (Jiangsu, China). For the pharmacokinetic assessment of zaltoprofen formulations, plasma samples were collected from the volunteers enrolled in the study.

### Preparation of stock solutions

Stock solutions of zaltoprofen and ketoprofen (IS) were prepared in methanol at the concentration of 1 mg/mL. These solutions were found to be stable for 10 h at room temperature and 31 d at -20°C.

Working solutions of zaltoprofen were prepared in methanol by appropriate dilution at a working concentration of 0.1, 0.5, 1, 5, 10, 50 and 80 µg/mL.

### Sample preparation and extraction procedure

A 1 mL aliquot of the collected plasma sample from a human volunteer was pipetted into a 10 mL centrifuge tube. The 50 µL working IS solution (1 mg/mL), and 50 µL hydrochloric acid solution (1 mol/L) were added and then vortexed for 10 s. Then, 5 mL diethyl ether were added again and vortexed for 1 min. After centrifugation of the samples at 4, 000 *g* for 10 min at room temperature, the organic layer was transferred to another 10 mL centrifuge tube and evaporated to dryness in a water bath at 40°C. The residue was redissolved in 100 µL methanol. After vortex and centrifugation, an aliquot of 20 µL was injected into the HPLC system.

### Standard curves and quality-control samples

Calibration curves were prepared by spiking blank plasma with proper volume of one of the above-mentioned working solutions to produce the standard curve points equivalent to 0.1, 0.5, 1, 5, 10, 50 and 80 µg/mL of zaltoprofen. Each sample also contained 50 µg/mL of the IS. The following assay procedures were the same as described above. In each run, a blank plasma sample (no IS) was also analyzed. Calibration curves were prepared by concentration *vs* the best fit of peak-area ratios (peak area of analyte/peak area of IS), and fitted to the equation (*y* = *bx+a*) by unweighted least-square regression.

Quality-control (QC) samples were prepared daily by spiking different samples of 1 mL blank plasma each with proper volume of the corresponding standard solution to produce a final concentration equivalent to low level (0.2 µg/mL), middle level (6.0 µg/mL) and high level (60 µg/mL) of zaltoprofen with 50 µg/mL of IS each. The following procedures were the same as described above.

### Method validation

The method validation assays were carried out following the US Food and Drug Administration (FDA) bioanalytical method validation guidance[6].

The method's specificity was tested by analyzing blank plasma samples of the healthy human subjects obtained from six different sources.

Standard curves of seven concentrations of zaltoprofen ranging 0.1-80 µg/mL were extracted and assayed. Blank plasma samples were analyzed to confirm absence of interferences, but were not used to construct the calibration function. The lower limit of quantification (LLOQ) was the lowest concentration of the standard curves. Each concentration standard should meet the following acceptable criteria: no more than 20% deviation at LLOQ and no more than 15% deviation for the standards above LLOQ.

The precision and accuracy of the assay were determined from the QC plasma samples by replicate analyses of three concentration levels of zaltoprofen (0.2, 6.0 and 60 µg/mL).

Within-batch precision and accuracy were determined by repeated analyses of five samples of zaltoprofen at each QC level (0.2, 6.0 and 60 µg/mL) on the same day (*n* = 5). Between batch precision and accuracy were determined by repeated analyses on three consecutive days (*n* = 5 per day). The concentration of each sample was determined using the standard curves prepared and analyzed on the same day.

The absolute extraction recovery of zaltoprofen was determined by comparson of the zaltoprofen /IS peak-area ratios obtained from extracted plasma samples with those from the standard solutions at the same concentration. This procedure was repeated for the three different concentration levels of 0.2, 6.0 and 60 µg/mL.

Freeze and thaw stability: Three concentration levels of QC plasma samples were tested after each of three freeze (-20°C)-thaw (room temperature) cycles.

Short-term temperature stability: Three concentration levels of QC plasma samples were kept at room temperature for a period that exceeded the routine preparation time of samples (around 8 h).

Long-term stability: Three concentration levels of QC plasma samples kept at low temperature (-20°C) were studied for a period of 15 and 29 d.

Postpreparative stability: The postpreparative stability was conducted by analyzing extracted QC samples kept at the room temperature for 24 h.

Stock solution stability: The stability of zaltoprofen and IS working solutions was evaluated at room temperature for 10 h and at low temperature (-20°C) for 31 d.

A standard curve in each analytical run was used to calculate the concentration of zaltoprofen in the run. It was prepared along with the unknown samples in the same batch.

### Pharmacokinetic analysis

Single- and multiple-dose pharmacokinetic parameters were calculated from plasma concentration-time data by noncompartmental methods. The maximal plasma concentration (C_max_) and the peak time (T_max_) were obtained from observed data. The area under the plasma concentration versus time curve (AUC) from 0 to the last measurable concentration (Ct), AUC_0-24_, was calculated by the linear trapezoidal rule. The area under the plasma concentration versus time curve from 0 to infinity (AUC_0-∞_) was calculated as AUC_0-24_ + C_t_/λ_z_, where λ_z_ is the slope of the log-linear regression of the terminal concentration data points. The terminal elimination half-life (t_1/2_) was calculated as (ln2)/λ_z_. The mean steady-state concentration (C_ss_) was calculated using AUC_ss(0-τ)_/τ, where τ is the dosing interval.

### Statistical analysis

Pharmacokinetic parameters were compared among dose levels using analysis of variance (ANOVA). The linearity of zaltoprofen pharmacokinetics was assessed by examining C_max_, AUC, and t_1/2_, as a function of the single-dose administration. Prior to comparisons, dose-dependent parameters (C_max_ and AUC) were normalized to the lowest dose. Data were presented as mean±standard deviation (SD). Paired Student *t* tests and a significance level of 5% were applied. The Drug and Statistics Software (DAS, Version 2.1.1; Mathematical Pharmacology Professional Committee of China, China) was used.

## RESULTS

### Method validation

**Fig.2:**
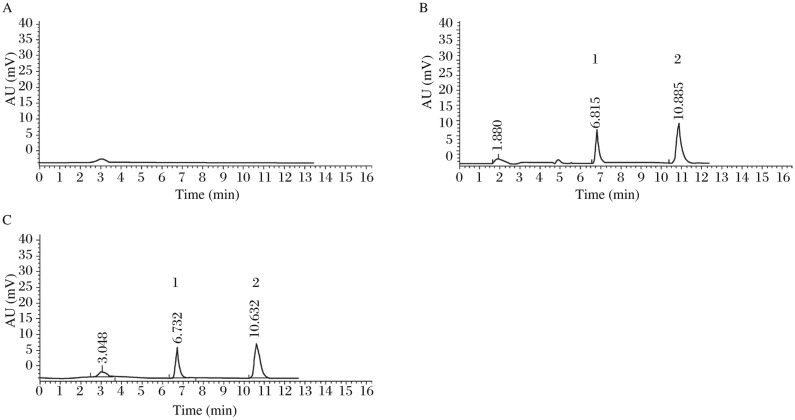
Chromatograms of zaltoprofen. A: in blank plasma. B: in plasma spiked with zaltoprofen (5.07 µg/mL) and IS. C: in plasma sample obtained at 2 h after a single oral dose of 160 mg zaltoprofen; IS (peak 1) and zaltoprofen (peak 2). The retention time of zaltoprofen and the IS was about 10.8 and 6.8 min, respectively. The blank plasma sample indicated no endogenous peaks at the retention positions of zaltoprofen or IS.

***Fig. 2A*** shows an HPLC chromatogram of a blank plasma sample indicating no endogenous peaks at the retention positions of zaltoprofen or IS. ***Fig. 2B*** and ***2C*** shows the typical chromatograms of a blank, spiked plasma sample with zaltoprofen (5.07 µg/mL), and a plasma sample obtained at 2 h from a subject who received a single oral dose (160 mg). The retention time of zaltoprofen and the IS was about 10.8 and 6.8 min, respectively.

The method was validated and found to be linear over the concentration range of 0.1-80 µg/mL (*r* = 0.9998) using (1/C^2^) weighted least squares regression. The relative standard deviation (RSD) values for within-batch precision and accuracy were in the range 3.07%-7.12% and 101.61%-103.75%, respectively, whereas the corresponding between-batch values were 3.75%-7.97% and 100.84%-102.48% (***Table 1***). The mean recoveries of the three concentration levels (0.2, 6.0 and 60 mg/mL) were 71.67%, 72.85% and 80.81%, respectively. The stability study showed that zaltoprofen and IS were stable in plasma for at least 8 h at room temperature and 29 d at –20°C. They were also stable at room temperature for at least 24 h after postpreparation as well as at –20°C after 3 freeze-thaw cycles (***Table 2***).

**Table 1:** The within- and between-batch precision and accuracy of the method

Added concentration (µg/mL)	Within-batch*				Between-batch^#^		
	Detected concentration (µg/mL)		Accuracy (%)	RSD (%)	Detected concentration (µg/mL)	Accuracy (%)	RSD (%)
0.20	0.21 ± 0.02		103.75	7.12	0.20 ± 0.02	100.84	7.97
6.08	6.18 ± 0.19		101.61	3.07	6.15 ± 0.23	101.15	3.75
60.78	62.48 ± 2.61		102.80	4.18	62.29 ± 4.58	102.48	4.59

*n = 5; ^#^*n* = 5 series per day; RSD: relative standard deviation.

**Table 2:** The stability of zaltoprofen in human plasma at different levels

	Accuracy		
	0.20 (µg/mL)	6.08 (µg/mL)	60.78 (µg/mL)
Short-term stability (8 h, room temperature)	101.09 ± 10.44	105.86 ± 4.24	105.19 ± 4.79
Long-term stability (15 d, -20°C)	107.29 ± 7.10	107.93 ± 4.51	98.18 ± 3.24
Long-term stability (29 d, -20°C)	95.48 ± 9.49	104.01 ± 7.79	97.47 ± 6.64
Freeze and thaw stability (1 cycle, -20°C, room temperature)	108.72 ± 13.45	109.22 ± 5.41	94.82 ± 3.02
Freeze and thaw stability (2 cycles, -20°C, room temperature)	97.29 ± 12.58	103.36 ± 9.71	88.71 ± 1.58
Freeze and thaw stability (3 cycles, -20°C, room temperature)	100.42 ± 11.31	105.25 ± 1.59	102.81 ± 6.46
Postpreparative stability (24 h, room temperature)	100.59 ± 6.60	100.11 ± 7.26	93.04 ± 2.54

(*n* = 5)

### Pharmacokinetics

**Fig.3:**
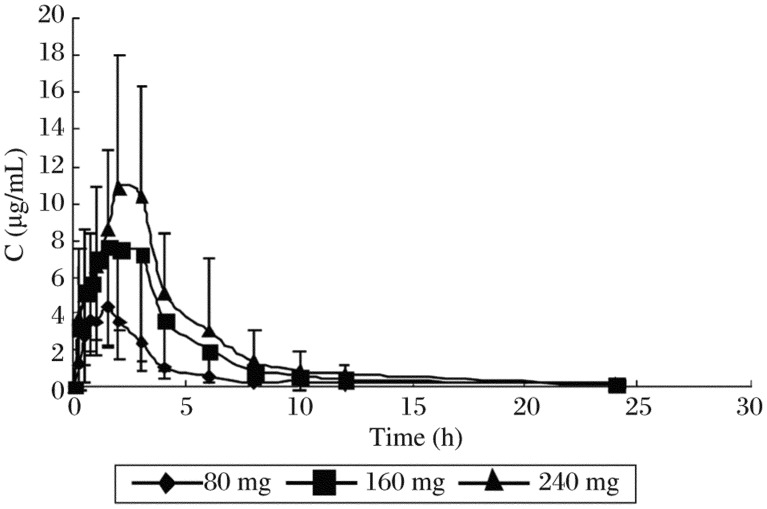
Mean concentration-time curves of zaltoprofen in 12 volunteers after oral administration of zaltoprofen in single-dose study (80, 160, and 240 mg). The plasma concentration increased in a proportional manner with the increasing single-dose of 80,160 and 240 mg.

**Fig.4:**
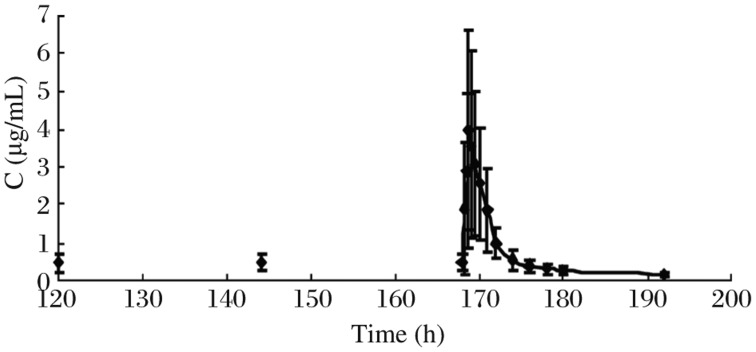
Mean concentration-time curves of zaltoprofen after oral administration of zaltoprofen in multiple doses (80 mg).

The method was applied to analyze plasma samples obtained from 12 healthy volunteers who received a single dose of 80, 160 and 240 mg zaltoprofen each and administered 80 mg zaltoprofen subsequently to achieve steady states in the pharmacokinetic study. The procedure developed was sensitive enough to assure the quantitative analysis of zaltoprofen in plasma with acceptable accuracy over a period of 24 h after a single oral administration. The mean plasma concentration–time curve of 12 volunteers after oral administration of zaltoprofen is shown in ***Fig. 3***. The mean plasma concentration-time profile following 3 times a day oral dosing of zaltoprofen is shown in ***Fig. 4***. Kinetic parameters are listed in ***Table 3*** and ***Table 4***. The results show that the t_1/2_ and the T_max_ did not change noticeably with the dose. The t_1/2_ and the T_max_ of consecutive administrations of 80 mg zaltoprofen were similar to those of single-dose of 80 mg zaltoprofen.

**Table 3:** Pharmacokinetic parameters of 12 healthy volunteers after oral administration of zaltoprofen tablets in single-dose study (80, 160 and 240 mg) and in multidose study (80 mg)

Parameters	80 mg	160 mg	240 mg	80 mg (steady states)
t_1/2_ (h)	4.83 ± 2.85	5.04 ± 2.10	4.83 ± 2.01	6.49 ± 4.05
T_max_(h)	1.46 ± 0.83	1.85 ± 0.93	2.33 ± 1.35	1.98 ± 2.03
C_max_(µg/mL)	5.37 ± 1.73	12.32 ± 6.66	16.41 ± 5.66	4.90 ± 2.30
AUC_0-24_(h·µg/mL)	14.93 ± 5.74	40.66 ± 24.43	52.19 ± 17.74	15.27 ± 5.66
AUC_0-∞_(h·µg/mL)	15.57 ± 5.79	41.61 ± 24.32	53.41 ± 18.10	16.54 ± 6.78

**Table 4:** Main pharmacokinetic parameters of zaltoprofen following multiple oral doses of zaltoprofen 80 mg 3 times daily

Parameters	80 mg 3 times daily
C^ss^_max_(µg/mL)	4.90 ± 2.30
C^ss^_min_(µg/mL)	0.46 ± 0.20
C_ss_(µg/mL)	1.49 ± 0.54
AUC_ss_(h·µg/mL)	11.89 ± 4.34

C^ss^_max_: maximal concentration of steady stauts; C^ss^_min_: minimal concentration of steady status. (*n* = 12)

The correlation analysis of dose and C_max_ showed that there was good linear response of different doses and C_max_. The linear regression equation was *C* = 0.069×*d* + 0.33, *r* = 0.99, where *d* corresponded to the dose and *C* refered to the C_max_ of three dose groups of zaltoprofen.

The correlation analysis of dose and AUC_0-24_ showed that there was a linear response of different doses and AUC. The linear regression equation was *A* = 0.233×*d* –1.33, *r* = 0.98, where *d* corresponded to the dose and *A* refered to the AUC_0-24_ of three dose groups of zaltoprofen.

Through one-way ANOVA of the main pharmacokinetic parameters between male and female volunteers after oral administration of zaltoprofen, C_max_ and AUC_0-24_ of three dose groups of male and female volunteers showed no significant difference (*P* > 0.05).

Single and multiple doses of zaltoprofen were well tolerated. There were no withdrawals and no adverse events in this study.

## DISCUSSION

Zaltoprofen is a NSAID with anti-inflammatory and analgesic effects on inflammatory pain[2], and it possesses novel anti-nociceptive mechanism by inhibiting B2-type bradykinin (BK) receptor function in nerve endings and selectively inhibiting PGE2 production at inflammatory sites and exhibits a powerful anti-inflammatory effect with a good safety margin. and a more strong inhibitory effect on BK-nociception than other NSAIDs[4].

However, little information has been reported on the pharmacokinetics of zaltoprofen in humans. So the study was performed to investigate the pharmacokinetic characteristics and dose proportionality of zaltoprofen in healthy Chinese volunteers following single oral doses of 80, 160, 240 mg and multiple oral doses of zaltoprofen 80 mg 3 times daily for 6 consecutive days.

Sample preparation is usually required for the determination of pharmaceuticals in biological samples owing to complex matrices in order to remove possibly interfering matrix components and increase the selectivity and sensitivity. Liquid–liquid extraction (LLE) is a widely adopted method and often achieves satisfactory extraction recoveries of analytes from biological samples. Dichloromethane, ethyl acetate, and diethyl ether were all tested. Diethyl ether finally chosen as the extraction solvent can not only eliminate the interference of endogenous substances, but also meet the requirement of sensitivity for the assay. For zaltoprofen is a weak acid, the extraction recovery of zaltoprofen can be improved by decreasing the pH of the plasma samples with hydrochloric acid solutions. Therefore, a 50 µL aliquot of 1 mol/L hydrochloric acid solution was added to 1 mL plasma sample before extraction. IS was applied to get high accuracy when zaltoprofen was determined in human plasma. Enalapril maleate, ibuprofen, fenbufen and ketoprofen were all investigated to find the suitable one. Ketoprofen was finally chosen as the IS because of its similarity of structure, retention time, and extraction efficiency as well as less endogenous interference in plasma. This suitable IS was useful to correct volumetric errors and to improve linearity and selectivity of the detection system. In the mobile phase, 10 mmol/L ammonium acetate buffer was added to improve the chromatographic peak shapes of zaltoprofen and IS, and obtain a good separation of target compounds.

The results of the study showed that zaltoprofen was rapidly absorbed following single oral doses, with maximum plasma concentrations occurring 1.46 to 2.33 h after administration. Mean t_1/2_ ranged from 4.83 to 5.04 h in the subjects. The C_max_ of zaltoprofen increased in a linear and proportional manner with increasing oral doses. The AUC and dose are also positively correlated. The t_1/2_ and the T_max_ did not change largely with the doses. In comparing the single-dose with the multiple-dose profile, the pharmacokinetic analysis of zaltoprofen showed similar properties. The t_1/2_ and the T_max_ of consecutive administrations of 80 mg zaltoprofen were similar to those of single-dose 80 mg zaltoprofen,which means that there is no significant accumulation of zaltoprofen with repeated doses. The linear pharmacokinetics and the low pharmacokinetic variability should facilitate the definition of dose regimens and the prediction of plasma levels after multiple oral administrations. As another main objective of the study, we used these data to examine the effects of sex on the pharmacokinetics of zaltoprofen. In both study parts, there were no significant differences in AUC and C_max_ between the female and male subjects. Hence, no adjustment of dosage on the basis of sex appears needed. Though the body weight of male and female volunteers have significant difference, we need not adjust dose according to body weight.

In conclusion, the results of the present study indicate that zaltoprofen has predictable pharmacokinetics. Zaltoprofen displays linear pharmacokinetics in the dose range of 80 to 240 mg after single oral doses. The compound is rapidly absorbed and cleared from plasma, which supports the use of zaltoprofen for a 3 times daily dose regimen. Zaltoprofen was not accumulated in the human body. No marked effect of sex on the disposition of zaltoprofen was observed. All these conclusions can provide reference for clinical medication.

Lee *et al*[5]. described the pharmacokinetic profiles of Korean healthy volunteers after oral administration of 160 mg zaltoprofen in single-dose study. The pharmacokinetic parameters of zaltoprofen were generally similar between Korean and Chinese volunteers.
